# 12-Eth­oxy-2,3,8,9-tetra­methoxy­benzo[*c*]phenanthridine dichloro­methane solvate

**DOI:** 10.1107/S1600536809050818

**Published:** 2009-12-12

**Authors:** Michael Ronaldson, Chad R. Maheux, James M. Nyangulu, T. Stanley Cameron, Manuel A.S. Aquino

**Affiliations:** a Department of Chemistry, St Francis Xavier University, PO Box 5000, Antigonish, Nova Scotia, Canada B2G 2W5; b Department of Chemistry, Dalhousie University, Halifax, Nova Scotia, Canada B3H 4R2

## Abstract

The title compound, C_23_H_23_NO_5_·CH_2_Cl_2_, was obtained *via* the alkyl­ation of the 12-hydr­oxy-2,3,8,9-tetra­methoxy­benzo[*c*]phenanthridine salt. The benzo[*c*]phenanthridine ring system is essentially planar, with a mean out-of-plane deviation of 0.026 Å. A dicloromethane mol­ecule of solvation is present and located between the sheets of phenanthridine mol­ecules, preventing any significant inter­molecular hydrogen-bonding or π–π inter­actions.

## Related literature

For related structures, see: Marek *et al.* (2002 ▶); Olugbade & Waigh (1996 ▶); Shabashov & Daugulis (2007 ▶).
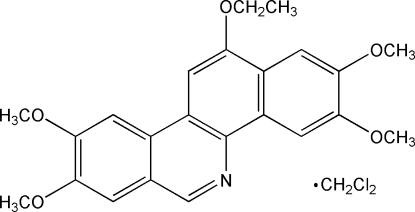


## Experimental

### 

#### Crystal data

C_23_H_23_NO_5_·CH_2_Cl_2_
*M* *_r_* = 478.37
Triclinic, 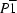

*a* = 7.6176 (7) Å
*b* = 12.874 (3) Å
*c* = 13.009 (2) Å
α = 107.468 (9)°
β = 96.7300 (11)°
γ = 103.134 (6)°
*V* = 1161.3 (3) Å^3^
*Z* = 2
Mo *K*α radiation
μ = 0.32 mm^−1^
*T* = 297 K
0.45 × 0.32 × 0.28 mm

#### Data collection

Rigaku R-AXIS RAPID diffractometer
Absorption correction: multi-scan (*ABSCOR*; Higashi, 1995 ▶) *T*_min_ = 0.735, *T*_max_ = 0.916
16719 measured reflections
4984 independent reflections
3144 reflections with *F* ^2^ > 2.0σ(*F* ^2^)
*R*_int_ = 0.042

#### Refinement

*R*[*F* ^2^ > 2σ(*F* ^2^)] = 0.056
*wR*(*F* ^2^) = 0.071
*S* = 1.10
3144 reflections
299 parameters
All H-atom parameters refined
Δρ_max_ = 0.23 e Å^−3^
Δρ_min_ = −0.29 e Å^−3^

### 

Data collection: *PROCESS* (Rigaku/MSC and Rigaku, 2006 ▶); cell refinement: *PROCESS*; data reduction: *CrystalStructure* (Rigaku/MSC and Rigaku, 2006 ▶); program(s) used to solve structure: *SHELXS86* (Sheldrick, 2008 ▶); program(s) used to refine structure: *CRYSTALS* (Betteridge *et al.*, 2003 ▶); molecular graphics: *SHELXTL* (Sheldrick, 2008 ▶); software used to prepare material for publication: *CrystalStructure*.

## Supplementary Material

Crystal structure: contains datablocks global, I. DOI: 10.1107/S1600536809050818/lh2944sup1.cif

Structure factors: contains datablocks I. DOI: 10.1107/S1600536809050818/lh2944Isup2.hkl

Additional supplementary materials:  crystallographic information; 3D view; checkCIF report

## Figures and Tables

**Fig. 1.**
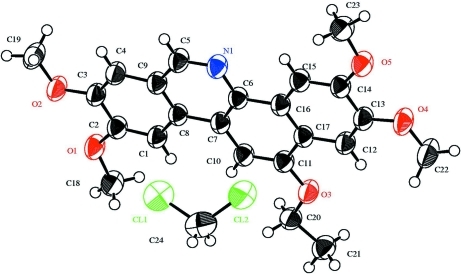
The molecular structure of (I), with atom labels and 50% probability displacement ellipsoids for the non-H atoms.

**Fig. 2.**
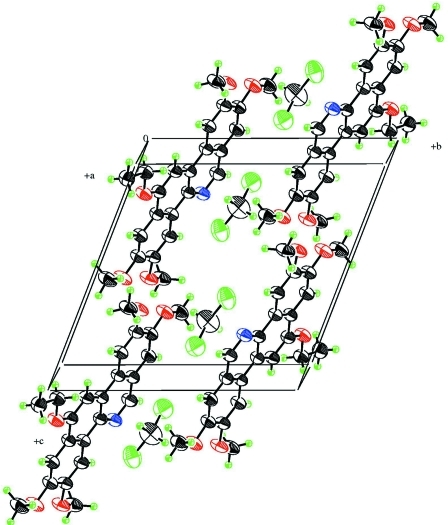
Part of the crystal structure of (I) viewed along the a axis, showing molecular chains along the c axis.

## supplementary crystallographic information

### Comment

Nitidine, a naturally occurring alkaloid and well known cytotoxic agent, has also proven to be substantially active against both chloroquine sensitive and chloroquine resistant strains of malaria (Olugbade & Waigh, 1996). The title compound is a nitidine analogue but does not contain a methylated heterocyclic nitrogen which makes it potentially suitable to coordinate to metal centres; a study we are currently undertaking. We report here the crystal structure of this nitidine analogue.

In the structure of the title compound (I) (Fig. 1) the key bond lengths (Table 1) are all similar to the related structures (Marek *et al.*, 2002 and Shabashov & Daugulis, 2007). The benzo[*c*]phenanthridine ring system is essentially planar with a mean out-of-plane deviation of 0.0256 Å with the largest deviation of 0.0538 (22) Å for atom C3.

No significant hydrogen bonding or π interactions are seen in the crystal packing of this compound presumably due to the presence of a dichloromethane molecule of solvation which is interspersed between planes of the parent molecule holding them far enough apart to prevent any interactions.

### Experimental

The 12-ethoxy-2,3,8,9-tetramethoxybenzo[*c*]phenanthridine was synthesized by the alkylation of the 12-hydroxy-2,3,8,9-tetramethoxybenzo[*c*]phenanthridine salt (Olugbade & Waigh, 1996). The 12-hydroxy-2,3,8,9-tetramethoxybenzo[*c*]phenanthridine salt (0.500 g, 1.08 mmol) and anhydrous potassium carbonate (0.442 g, 0.320 mmol) were added together in 20 ml of anhydrous acetone. The green-yellow mixture was refluxed with stirring for 30 minutes and iodoethane (0.258 ml, 0.2 mmol) was then added. The reflux was allowed to proceed for 2 h during which time the colour changed from green-yellow to light brown. The mixture was filtered and the solid washed with hot dichloromethane. The yellow filtrate was collected and concentrated. The orange-brown product was taken up in 200 ml of water and extracted with 200 ml of dichloromethane. The dichloromethane extract was concentrated and the resulting light cream coloured product was dried *in vacuo*. (Yield = 0.318 g, 75%).

### Refinement

Hydrogen atoms were refined using the riding model.

### Crystal data

**Table:**

C_23_H_23_NO_5_·CH_2_Cl_2_	*Z* = 2
*M**_r_* = 478.37	*F*(000) = 500.00
Triclinic, *P*1	*D*_x_ = 1.368 Mg m^−^^3^
Hall symbol: -P 1	Mo *K*α radiation, λ = 0.71070 Å
*a* = 7.6176 (7) Å	Cell parameters from 2532 reflections
*b* = 12.874 (3) Å	θ = 2.8–35.0°
*c* = 13.009 (2) Å	µ = 0.32 mm^−^^1^
α = 107.468 (9)°	*T* = 297 K
β = 96.7300 (11)°	Block, colourless
γ = 103.134 (6)°	0.45 × 0.32 × 0.28 mm
*V* = 1161.3 (3) Å^3^	

### Data collection

**Table:**

Rigaku R-AXIS RAPID diffractometer	3144 reflections with *F*^2^ > 2.0σ(*F*^2^)
Detector resolution: 10.00 pixels mm^-1^	*R*_int_ = 0.042
ω scans	θ_max_ = 35.7°
Absorption correction: multi-scan (*ABSCOR*; Higashi, 1995)	*h* = −12→12
*T*_min_ = 0.735, *T*_max_ = 0.916	*k* = −21→21
16719 measured reflections	*l* = −21→21
4984 independent reflections	

### Refinement

**Table:**

Refinement on *F*	0 restraints
*R*[*F*^2^ > 2σ(*F*^2^)] = 0.056	All H-atom parameters refined
*wR*(*F*^2^) = 0.071	Chebychev polynomial *w*ith 3 parameters (Carruthers & Watkin, 1979) 9.5043 4.1025 7.0826
*S* = 1.10	(Δ/σ)_max_ < 0.001
3144 reflections	Δρ_max_ = 0.23 e Å^−^^3^
299 parameters	Δρ_min_ = −0.29 e Å^−^^3^

### Special details

**Table:**

Refinement. Refinement was performed using reflections with *F*^2^ > 3.0 σ(*F*^2^). The weighted *R*-factor(*wR*), goodness of fit (*S*) and *R*-factor (gt) are based on *F*, with *F* set to zero for negative *F*. The threshold expression of *F*^2^ > 2.0 σ(*F*^2^) is used only for calculating *R*-factor (gt).

### Fractional atomic coordinates and isotropic or equivalent isotropic displacement parameters (Å^2^)

**Table:**

	*x*	*y*	*z*	*U*_iso_*/*U*_eq_	
CL1	0.57834 (14)	0.48531 (8)	0.12892 (8)	0.1030 (3)	
CL2	0.65852 (14)	0.43659 (8)	0.33198 (9)	0.1048 (3)	
O1	0.4347 (2)	0.23338 (16)	−0.29456 (13)	0.0659 (4)	
O2	0.7420 (2)	0.34392 (16)	−0.31719 (13)	0.0656 (4)	
O3	0.3983 (2)	0.07039 (14)	0.19755 (12)	0.0597 (4)	
O4	0.8982 (2)	0.12439 (17)	0.51431 (14)	0.0720 (5)	
O5	1.1964 (2)	0.23385 (16)	0.48175 (13)	0.0674 (4)	
N1	1.0471 (2)	0.31310 (17)	0.12497 (15)	0.0568 (5)	
C1	0.5648 (3)	0.2258 (2)	−0.11897 (17)	0.0538 (5)	
C2	0.5768 (3)	0.25700 (19)	−0.21031 (17)	0.0533 (5)	
C3	0.7499 (3)	0.3197 (2)	−0.22228 (17)	0.0539 (5)	
C4	0.9017 (3)	0.3493 (2)	−0.14131 (18)	0.0551 (6)	
C5	1.0462 (3)	0.3431 (2)	0.03733 (18)	0.0582 (6)	
C6	0.8815 (2)	0.25253 (18)	0.13772 (16)	0.0486 (5)	
C7	0.7164 (2)	0.22220 (18)	0.06207 (17)	0.0492 (5)	
C8	0.7207 (2)	0.25445 (18)	−0.03513 (16)	0.0485 (5)	
C9	0.8894 (3)	0.31686 (18)	−0.04683 (17)	0.0516 (5)	
C10	0.5508 (2)	0.16038 (19)	0.08156 (17)	0.0526 (5)	
C11	0.5507 (2)	0.12897 (18)	0.17248 (17)	0.0504 (5)	
C12	0.7184 (3)	0.1245 (2)	0.34684 (18)	0.0543 (5)	
C13	0.8790 (3)	0.1520 (2)	0.42088 (17)	0.0562 (6)	
C14	1.0466 (3)	0.2147 (2)	0.40409 (17)	0.0535 (5)	
C15	1.0454 (3)	0.24772 (19)	0.31279 (18)	0.0536 (5)	
C16	0.8821 (3)	0.21968 (18)	0.23475 (16)	0.0494 (5)	
C17	0.7176 (2)	0.15731 (18)	0.25179 (16)	0.0495 (5)	
C18	0.2535 (3)	0.1832 (2)	−0.2814 (2)	0.0682 (7)	
C19	0.9107 (3)	0.4051 (2)	−0.3347 (2)	0.0717 (7)	
C20	0.2253 (3)	0.0407 (2)	0.12298 (18)	0.0566 (6)	
C21	0.0823 (3)	−0.0271 (2)	0.1655 (2)	0.0653 (7)	
C22	0.7374 (4)	0.0634 (2)	0.5381 (2)	0.0810 (8)	
C23	1.3707 (3)	0.2858 (2)	0.4631 (2)	0.0709 (7)	
C24	0.5047 (5)	0.4727 (4)	0.2476 (3)	0.1141 (15)	
H1	0.4493	0.1844	−0.1119	0.066*	
H2	1.0164	0.3915	−0.1485	0.091*	
H3	1.1598	0.3859	0.0292	0.076*	
H4	0.4388	0.1406	0.0303	0.063*	
H5	0.6073	0.0834	0.3590	0.068*	
H6	1.1569	0.2901	0.3021	0.060*	
H7	0.2534	0.1169	−0.2633	0.097*	
H8	0.1678	0.1631	−0.3484	0.088*	
H9	0.2196	0.2357	−0.2245	0.070*	
H10	0.9948	0.3601	−0.3420	0.088*	
H11	0.9624	0.4733	−0.2740	0.088*	
H12	0.8881	0.4229	−0.4001	0.088*	
H13	0.1944	0.1075	0.1204	0.068*	
H14	0.2328	−0.0034	0.0516	0.068*	
H15	1.3747	0.3604	0.4643	0.085*	
H16	1.3874	0.2426	0.3935	0.085*	
H17	1.4659	0.2889	0.5191	0.085*	
H18	−0.0328	−0.0504	0.1162	0.078*	
H19	0.0722	0.0184	0.2359	0.078*	
H20	0.1167	−0.0919	0.1712	0.078*	
H21	0.6854	−0.0066	0.4797	0.099*	
H22	0.7681	0.0493	0.6046	0.099*	
H23	0.6508	0.1066	0.5457	0.099*	
H24	0.4886	0.5435	0.2886	0.228*	
H25	0.3920	0.4160	0.2264	0.232*	

### Atomic displacement parameters (Å^2^)

**Table:**

	*U*^11^	*U*^22^	*U*^33^	*U*^12^	*U*^13^	*U*^23^
CL1	0.1017 (6)	0.1080 (6)	0.1040 (6)	0.0293 (5)	0.0281 (5)	0.0396 (5)
CL2	0.1012 (6)	0.1100 (6)	0.1307 (7)	0.0361 (5)	0.0482 (5)	0.0656 (5)
O1	0.0527 (9)	0.0983 (12)	0.0515 (8)	0.0100 (8)	0.0015 (7)	0.0434 (8)
O2	0.0567 (9)	0.0941 (11)	0.0543 (8)	0.0111 (8)	0.0100 (7)	0.0445 (8)
O3	0.0472 (8)	0.0802 (10)	0.0541 (8)	0.0057 (7)	0.0054 (6)	0.0366 (7)
O4	0.0577 (9)	0.1055 (13)	0.0588 (9)	0.0072 (9)	0.0015 (7)	0.0515 (9)
O5	0.0500 (8)	0.0993 (12)	0.0544 (8)	0.0082 (8)	−0.0008 (7)	0.0416 (8)
N1	0.0493 (10)	0.0692 (11)	0.0509 (9)	0.0070 (8)	0.0029 (7)	0.0286 (8)
C1	0.0485 (11)	0.0694 (13)	0.0479 (10)	0.0122 (9)	0.0076 (9)	0.0297 (10)
C2	0.0497 (11)	0.0662 (12)	0.0449 (10)	0.0116 (9)	0.0042 (8)	0.0253 (9)
C3	0.0547 (12)	0.0673 (13)	0.0460 (10)	0.0145 (10)	0.0109 (9)	0.0294 (10)
C4	0.0541 (12)	0.0649 (12)	0.0497 (11)	0.0113 (10)	0.0105 (9)	0.0279 (10)
C5	0.0513 (12)	0.0714 (13)	0.0510 (11)	0.0060 (10)	0.0041 (9)	0.0300 (10)
C6	0.0472 (10)	0.0551 (11)	0.0430 (10)	0.0102 (8)	0.0044 (8)	0.0204 (8)
C7	0.0497 (11)	0.0563 (11)	0.0450 (10)	0.0141 (9)	0.0070 (8)	0.0230 (9)
C8	0.0491 (11)	0.0570 (11)	0.0420 (9)	0.0134 (9)	0.0067 (8)	0.0224 (9)
C9	0.0505 (11)	0.0591 (11)	0.0469 (10)	0.0119 (9)	0.0066 (8)	0.0238 (9)
C10	0.0470 (11)	0.0650 (12)	0.0468 (10)	0.0115 (9)	0.0033 (8)	0.0254 (9)
C11	0.0460 (11)	0.0582 (11)	0.0470 (10)	0.0103 (9)	0.0055 (8)	0.0223 (9)
C12	0.0489 (11)	0.0662 (12)	0.0515 (11)	0.0109 (9)	0.0075 (9)	0.0298 (10)
C13	0.0564 (12)	0.0700 (13)	0.0473 (11)	0.0152 (10)	0.0085 (9)	0.0293 (10)
C14	0.0504 (11)	0.0677 (13)	0.0434 (10)	0.0133 (9)	0.0037 (8)	0.0245 (9)
C15	0.0503 (11)	0.0628 (12)	0.0479 (11)	0.0112 (9)	0.0046 (8)	0.0244 (9)
C16	0.0508 (11)	0.0573 (11)	0.0433 (10)	0.0150 (9)	0.0075 (8)	0.0223 (9)
C17	0.0496 (11)	0.0570 (11)	0.0448 (10)	0.0141 (9)	0.0058 (8)	0.0232 (9)
C18	0.0452 (12)	0.0979 (18)	0.0603 (13)	0.0052 (11)	−0.0021 (10)	0.0404 (13)
C19	0.0683 (15)	0.0919 (17)	0.0630 (14)	0.0114 (13)	0.0199 (12)	0.0430 (13)
C20	0.0478 (11)	0.0678 (13)	0.0546 (12)	0.0120 (10)	0.0080 (9)	0.0249 (10)
C21	0.0566 (13)	0.0704 (14)	0.0673 (14)	0.0082 (10)	0.0135 (11)	0.0276 (12)
C22	0.0703 (16)	0.111 (2)	0.0660 (15)	0.0031 (14)	0.0095 (12)	0.0538 (15)
C23	0.0503 (12)	0.105 (2)	0.0566 (13)	0.0118 (12)	0.0015 (10)	0.0363 (13)
C24	0.085 (2)	0.157 (3)	0.128 (3)	0.049 (2)	0.044 (2)	0.069 (2)

### Geometric parameters (Å, °)

**Table:**

CL1—C24	1.743 (5)	C14—C15	1.376 (3)
CL2—C24	1.746 (5)	C15—C16	1.410 (3)
O1—C2	1.355 (2)	C16—C17	1.407 (3)
O1—C18	1.439 (3)	C20—C21	1.501 (3)
O2—C3	1.359 (3)	C1—H1	0.949
O2—C19	1.429 (3)	C4—H2	0.950
O3—C11	1.365 (2)	C5—H3	0.950
O3—C20	1.439 (2)	C10—H4	0.950
O4—C13	1.366 (3)	C12—H5	0.948
O4—C22	1.418 (3)	C15—H6	0.950
O5—C14	1.355 (2)	C18—H7	0.951
O5—C23	1.427 (3)	C18—H8	0.953
N1—C5	1.309 (3)	C18—H9	0.946
N1—C6	1.381 (2)	C19—H10	0.951
C1—C2	1.370 (3)	C19—H11	0.950
C1—C8	1.412 (2)	C19—H12	0.951
C2—C3	1.434 (3)	C20—H13	0.950
C3—C4	1.366 (3)	C20—H14	0.947
C4—C9	1.418 (3)	C21—H18	0.950
C5—C9	1.424 (3)	C21—H19	0.950
C6—C7	1.400 (2)	C21—H20	0.950
C6—C16	1.446 (3)	C22—H21	0.950
C7—C8	1.446 (3)	C22—H22	0.950
C7—C10	1.424 (3)	C22—H23	0.950
C8—C9	1.403 (3)	C23—H15	0.950
C10—C11	1.360 (3)	C23—H16	0.950
C11—C17	1.439 (2)	C23—H17	0.950
C12—C13	1.368 (3)	C24—H24	0.951
C12—C17	1.422 (3)	C24—H25	0.941
C13—C14	1.425 (3)		
			
CL1···CL1^i^	3.5843 (15)	H8···O4^xi^	3.566
CL1···C8	3.573 (2)	H8···O5^iv^	2.644
CL2···C16	3.584 (2)	H8···C13^iv^	3.453
CL2···C17	3.579 (2)	H8···C14^iv^	3.540
CL2···C19^ii^	3.436 (2)	H8···C22^iv^	3.224
CL2···C23^iii^	3.524 (3)	H8···C22^xi^	3.488
O1···O5^iv^	3.247 (2)	H8···H10^iii^	3.098
O1···C23^iv^	3.420 (3)	H8···H17^iv^	3.417
O1···C24^i^	3.557 (5)	H8···H19^xiii^	3.417
O2···O4^v^	3.554 (2)	H8···H20^xiii^	3.520
O2···C22^v^	3.526 (3)	H8···H21^xi^	2.762
O2···C23^iv^	3.547 (3)	H8···H22^iv^	2.965
O2···C24^i^	3.325 (5)	H9···CL1^i^	3.343
O4···O2^vi^	3.554 (2)	H9···C4^iii^	3.232
O4···C18^vii^	3.338 (2)	H9···C21^xiii^	3.454
O4···C19^vi^	3.531 (3)	H9···H2^iii^	2.824
O5···O1^vii^	3.247 (2)	H9···H3^iii^	3.429
O5···C18^vii^	3.337 (3)	H9···H10^iii^	3.153
N1···C24^viii^	3.520 (3)	H9···H18^xiii^	3.261
C8···CL1	3.573 (2)	H9···H19^xiii^	3.462
C15···C21^viii^	3.574 (3)	H9···H20^xiii^	3.082
C16···CL2	3.584 (2)	H9···H24^i^	3.565
C17···CL2	3.579 (2)	H10···CL2^ii^	3.227
C18···O4^iv^	3.338 (2)	H10···O4^v^	2.916
C18···O5^iv^	3.337 (3)	H10···O5^v^	3.159
C19···CL2^ii^	3.436 (2)	H10···C13^v^	3.286
C19···O4^v^	3.531 (3)	H10···C14^v^	3.391
C21···C15^iii^	3.574 (3)	H10···C18^viii^	3.528
C22···O2^vi^	3.526 (3)	H10···H8^viii^	3.098
C22···C22^ix^	3.498 (3)	H10···H9^viii^	3.153
C23···CL2^viii^	3.524 (3)	H10···H23^v^	3.465
C23···O1^vii^	3.420 (3)	H11···CL2^ii^	3.125
C23···O2^vii^	3.547 (3)	H11···N1^ii^	2.872
C24···O1^i^	3.557 (5)	H11···C5^ii^	3.289
C24···O2^i^	3.325 (5)	H11···C6^ii^	3.295
C24···N1^iii^	3.520 (3)	H11···H3^ii^	3.506
CL1···H2^ii^	3.075	H11···H6^ii^	3.476
CL1···H3^iii^	3.109	H11···H24^i^	3.372
CL1···H3^ii^	3.531	H11···H25^i^	3.364
CL1···H9^i^	3.343	H12···CL2^ii^	3.386
CL2···H10^ii^	3.227	H12···C13^v^	3.545
CL2···H11^ii^	3.125	H12···H17^iv^	3.166
CL2···H12^ii^	3.386	H12···H24^i^	3.419
CL2···H15^iii^	3.086	H13···N1^iii^	3.089
CL2···H15^x^	3.196	H13···C6^iii^	3.334
CL2···H16^iii^	3.222	H13···C15^iii^	3.114
O1···H17^iv^	2.746	H13···C16^iii^	3.325
O1···H21^xi^	3.034	H13···H6^iii^	2.885
O1···H23^v^	3.111	H13···H14^xiii^	3.450
O1···H24^i^	2.773	H13···H16^iii^	3.422
O2···H17^iv^	2.613	H13···H18^xiii^	2.979
O2···H23^v^	2.911	H14···C6^xi^	3.256
O2···H24^i^	2.512	H14···C7^xi^	2.899
O2···H25^i^	3.393	H14···C8^xi^	3.277
O3···H16^iii^	2.857	H14···C10^xi^	3.145
O3···H22^ix^	3.587	H14···H4^xi^	3.466
O4···H8^vii^	2.408	H14···H13^xiii^	3.450
O4···H8^xi^	3.566	H14···H18^xiii^	2.859
O4···H10^vi^	2.916	H15···CL2^viii^	3.086
O5···H8^vii^	2.644	H15···CL2^x^	3.196
O5···H10^vi^	3.159	H15···H15^xiv^	3.447
O5···H19^viii^	3.404	H15···H24^x^	3.031
O5···H21^xii^	3.404	H15···H25^viii^	3.389
O5···H22^xii^	3.561	H16···CL2^viii^	3.222
O5···H24^x^	3.598	H16···O3^viii^	2.857
N1···H11^ii^	2.872	H16···C11^viii^	3.331
N1···H13^viii^	3.089	H16···C12^viii^	3.254
N1···H25^viii^	2.637	H16···C17^viii^	3.473
C1···H20^xi^	3.303	H16···C20^viii^	3.564
C2···H20^xi^	3.586	H16···H5^viii^	2.897
C2···H23^v^	3.372	H16···H13^viii^	3.422
C2···H24^i^	3.149	H16···H19^viii^	3.232
C3···H20^xi^	3.528	H16···H25^viii^	3.550
C3···H22^v^	3.577	H17···O1^vii^	2.746
C3···H23^v^	3.281	H17···O2^vii^	2.613
C3···H24^i^	3.024	H17···C19^vii^	3.437
C4···H3^ii^	3.445	H17···C24^x^	3.552
C4···H9^viii^	3.232	H17···H5^viii^	3.318
C4···H20^xi^	3.187	H17···H8^vii^	3.417
C5···H2^ii^	3.453	H17···H12^vii^	3.166
C5···H11^ii^	3.289	H17···H21^xii^	3.559
C5···H20^xi^	3.388	H17···H23^viii^	3.081
C5···H25^viii^	3.137	H17···H24^x^	2.692
C6···H11^ii^	3.295	H18···C16^iii^	3.596
C6···H13^viii^	3.334	H18···C18^xiii^	3.479
C6···H14^xi^	3.256	H18···C20^xiii^	3.332
C7···H14^xi^	2.899	H18···H1^xiii^	3.234
C7···H20^xi^	3.529	H18···H4^xiii^	3.194
C8···H14^xi^	3.277	H18···H7^xiii^	2.881
C8···H20^xi^	2.918	H18···H9^xiii^	3.261
C9···H20^xi^	2.849	H18···H13^xiii^	2.979
C10···H14^xi^	3.145	H18···H14^xiii^	2.859
C11···H16^iii^	3.331	H19···O5^iii^	3.404
C12···H7^xi^	3.035	H19···C12^iii^	3.551
C12···H16^iii^	3.254	H19···C13^iii^	3.227
C12···H19^viii^	3.551	H19···C14^iii^	2.867
C13···H7^xi^	3.297	H19···C15^iii^	2.882
C13···H8^vii^	3.453	H19···C16^iii^	3.246
C13···H10^vi^	3.286	H19···C17^iii^	3.555
C13···H12^vi^	3.545	H19···C18^xiii^	3.403
C13···H19^viii^	3.227	H19···H6^iii^	3.229
C14···H8^vii^	3.540	H19···H7^xiii^	2.816
C14···H10^vi^	3.391	H19···H8^xiii^	3.417
C14···H19^viii^	2.867	H19···H9^xiii^	3.462
C15···H13^viii^	3.114	H19···H16^iii^	3.232
C15···H19^viii^	2.882	H19···H22^ix^	2.743
C15···H25^viii^	3.533	H20···C1^xi^	3.303
C16···H13^viii^	3.325	H20···C2^xi^	3.586
C16···H18^viii^	3.596	H20···C3^xi^	3.528
C16···H19^viii^	3.246	H20···C4^xi^	3.187
C17···H16^iii^	3.473	H20···C5^xi^	3.388
C17···H19^viii^	3.555	H20···C7^xi^	3.529
C18···H10^iii^	3.528	H20···C8^xi^	2.918
C18···H18^xiii^	3.479	H20···C9^xi^	2.849
C18···H19^xiii^	3.403	H20···C18^xiii^	3.441
C18···H20^xiii^	3.441	H20···H7^xiii^	3.178
C18···H21^xi^	3.046	H20···H8^xiii^	3.520
C19···H17^iv^	3.437	H20···H9^xiii^	3.082
C19···H24^i^	3.340	H20···H22^ix^	2.793
C20···H6^iii^	3.545	H21···O1^xi^	3.034
C20···H16^iii^	3.564	H21···O5^xii^	3.404
C20···H18^xiii^	3.332	H21···C18^xi^	3.046
C21···H7^xiii^	3.130	H21···C22^ix^	3.101
C21···H9^xiii^	3.454	H21···H5^ix^	3.419
C21···H22^ix^	3.193	H21···H7^xi^	2.892
C22···H5^ix^	3.514	H21···H8^xi^	2.762
C22···H8^vii^	3.224	H21···H17^xii^	3.559
C22···H8^xi^	3.488	H21···H21^ix^	2.967
C22···H21^ix^	3.101	H21···H22^ix^	3.365
C22···H23^ix^	3.097	H21···H23^ix^	2.525
C23···H5^viii^	3.530	H22···O3^ix^	3.587
C23···H24^x^	3.204	H22···O5^xii^	3.561
C24···H3^iii^	3.351	H22···C3^vi^	3.577
C24···H6^iii^	3.414	H22···C21^ix^	3.193
C24···H17^x^	3.552	H22···H5^ix^	3.147
H1···H18^xiii^	3.234	H22···H8^vii^	2.965
H2···CL1^ii^	3.075	H22···H19^ix^	2.743
H2···C5^ii^	3.453	H22···H20^ix^	2.793
H2···H3^ii^	3.462	H22···H21^ix^	3.365
H2···H9^viii^	2.824	H22···H23^ix^	3.362
H3···CL1^viii^	3.109	H23···O1^vi^	3.111
H3···CL1^ii^	3.531	H23···O2^vi^	2.911
H3···C4^ii^	3.445	H23···C2^vi^	3.372
H3···C24^viii^	3.351	H23···C3^vi^	3.281
H3···H2^ii^	3.462	H23···C22^ix^	3.097
H3···H9^viii^	3.429	H23···H5^ix^	3.405
H3···H11^ii^	3.506	H23···H10^vi^	3.465
H3···H25^viii^	2.809	H23···H17^iii^	3.081
H4···H14^xi^	3.466	H23···H21^ix^	2.525
H4···H18^xiii^	3.194	H23···H22^ix^	3.362
H5···C22^ix^	3.514	H23···H23^ix^	2.960
H5···C23^iii^	3.530	H24···O1^i^	2.773
H5···H7^xi^	2.991	H24···O2^i^	2.512
H5···H16^iii^	2.897	H24···O5^x^	3.598
H5···H17^iii^	3.318	H24···C2^i^	3.149
H5···H21^ix^	3.419	H24···C3^i^	3.024
H5···H22^ix^	3.147	H24···C19^i^	3.340
H5···H23^ix^	3.405	H24···C23^x^	3.204
H6···C20^viii^	3.545	H24···H9^i^	3.565
H6···C24^viii^	3.414	H24···H11^i^	3.372
H6···H11^ii^	3.476	H24···H12^i^	3.419
H6···H13^viii^	2.885	H24···H15^x^	3.031
H6···H19^viii^	3.229	H24···H17^x^	2.692
H6···H25^viii^	2.618	H25···O2^i^	3.393
H7···C12^xi^	3.035	H25···N1^iii^	2.637
H7···C13^xi^	3.297	H25···C5^iii^	3.137
H7···C21^xiii^	3.130	H25···C15^iii^	3.533
H7···H5^xi^	2.991	H25···H3^iii^	2.809
H7···H18^xiii^	2.881	H25···H6^iii^	2.618
H7···H19^xiii^	2.816	H25···H11^i^	3.364
H7···H20^xiii^	3.178	H25···H15^iii^	3.389
H7···H21^xi^	2.892	H25···H16^iii^	3.550
H8···O4^iv^	2.408		
			
C2—O1—C18	117.3 (2)	C9—C4—H2	119.8
C3—O2—C19	116.52 (18)	N1—C5—H3	117.2
C11—O3—C20	117.65 (18)	C9—C5—H3	117.4
C13—O4—C22	117.39 (19)	C7—C10—H4	119.6
C14—O5—C23	117.0 (2)	C11—C10—H4	119.5
C5—N1—C6	117.22 (18)	C13—C12—H5	119.8
C2—C1—C8	121.2 (2)	C17—C12—H5	119.9
O1—C2—C1	125.2 (2)	C14—C15—H6	119.3
O1—C2—C3	114.7 (2)	C16—C15—H6	119.3
C1—C2—C3	120.10 (19)	O1—C18—H7	109.3
O2—C3—C2	113.76 (18)	O1—C18—H8	109.5
O2—C3—C4	126.8 (2)	O1—C18—H9	109.5
C2—C3—C4	119.5 (2)	H7—C18—H8	109.2
C3—C4—C9	120.5 (2)	H7—C18—H9	109.7
N1—C5—C9	125.4 (2)	H8—C18—H9	109.5
N1—C6—C7	123.3 (2)	O2—C19—H10	109.6
N1—C6—C16	117.29 (18)	O2—C19—H11	109.6
C7—C6—C16	119.40 (19)	O2—C19—H12	109.6
C6—C7—C8	118.13 (19)	H10—C19—H11	109.3
C6—C7—C10	119.9 (2)	H10—C19—H12	109.3
C8—C7—C10	121.93 (18)	H11—C19—H12	109.4
C1—C8—C7	123.47 (19)	O3—C20—H13	109.9
C1—C8—C9	118.4 (2)	O3—C20—H14	110.1
C7—C8—C9	118.11 (18)	C21—C20—H13	109.9
C4—C9—C5	121.8 (2)	C21—C20—H14	109.8
C4—C9—C8	120.37 (19)	H13—C20—H14	109.7
C5—C9—C8	117.8 (2)	C20—C21—H18	109.4
C7—C10—C11	120.88 (19)	C20—C21—H19	109.4
O3—C11—C10	124.55 (18)	C20—C21—H20	109.6
O3—C11—C17	114.4 (2)	H18—C21—H19	109.5
C10—C11—C17	121.05 (19)	H18—C21—H20	109.5
C13—C12—C17	120.2 (2)	H19—C21—H20	109.5
O4—C13—C12	125.8 (2)	O4—C22—H21	109.4
O4—C13—C14	113.77 (19)	O4—C22—H22	109.6
C12—C13—C14	120.4 (2)	O4—C22—H23	109.5
O5—C14—C13	114.6 (2)	H21—C22—H22	109.5
O5—C14—C15	126.0 (2)	H21—C22—H23	109.5
C13—C14—C15	119.38 (19)	H22—C22—H23	109.5
C14—C15—C16	121.4 (2)	O5—C23—H15	109.4
C6—C16—C15	121.4 (2)	O5—C23—H16	109.4
C6—C16—C17	119.81 (18)	O5—C23—H17	109.6
C15—C16—C17	118.8 (2)	H15—C23—H16	109.5
C11—C17—C12	121.28 (19)	H15—C23—H17	109.5
C11—C17—C16	118.9 (2)	H16—C23—H17	109.5
C12—C17—C16	119.81 (18)	CL1—C24—H24	108.2
O3—C20—C21	107.5 (2)	CL1—C24—H25	108.2
CL1—C24—CL2	113.8 (2)	CL2—C24—H24	108.3
C2—C1—H1	119.3	CL2—C24—H25	108.3
C8—C1—H1	119.5	H24—C24—H25	110.2
C3—C4—H2	119.8		
			
C18—O1—C2—C1	7.6 (3)	C16—C6—C7—C8	−178.9 (2)
C18—O1—C2—C3	−172.5 (2)	C16—C6—C7—C10	0.5 (3)
C19—O2—C3—C2	−179.2 (2)	C6—C7—C8—C1	178.0 (2)
C19—O2—C3—C4	1.1 (3)	C6—C7—C8—C9	−1.6 (3)
C11—O3—C20—C21	177.49 (19)	C6—C7—C10—C11	−0.8 (3)
C20—O3—C11—C10	−0.5 (3)	C8—C7—C10—C11	178.6 (2)
C20—O3—C11—C17	178.95 (19)	C10—C7—C8—C1	−1.5 (3)
C22—O4—C13—C12	−1.2 (3)	C10—C7—C8—C9	179.0 (2)
C22—O4—C13—C14	179.1 (2)	C1—C8—C9—C4	−0.4 (3)
C23—O5—C14—C13	173.6 (2)	C1—C8—C9—C5	−178.7 (2)
C23—O5—C14—C15	−4.8 (3)	C7—C8—C9—C4	179.1 (2)
C5—N1—C6—C7	0.3 (3)	C7—C8—C9—C5	0.8 (3)
C5—N1—C6—C16	−179.8 (2)	C7—C10—C11—O3	179.6 (2)
C6—N1—C5—C9	−1.2 (3)	C7—C10—C11—C17	0.1 (2)
C2—C1—C8—C7	−179.0 (2)	O3—C11—C17—C12	0.6 (3)
C2—C1—C8—C9	0.5 (3)	O3—C11—C17—C16	−178.76 (19)
C8—C1—C2—O1	−179.93 (18)	C10—C11—C17—C12	−179.9 (2)
C8—C1—C2—C3	0.1 (3)	C10—C11—C17—C16	0.8 (3)
O1—C2—C3—O2	−0.6 (3)	C13—C12—C17—C11	179.7 (2)
O1—C2—C3—C4	179.1 (2)	C13—C12—C17—C16	−0.9 (3)
C1—C2—C3—O2	179.4 (2)	C17—C12—C13—O4	−179.2 (2)
C1—C2—C3—C4	−0.9 (3)	C17—C12—C13—C14	0.6 (3)
O2—C3—C4—C9	−179.3 (2)	O4—C13—C14—O5	1.5 (3)
C2—C3—C4—C9	1.0 (3)	O4—C13—C14—C15	−179.98 (16)
C3—C4—C9—C5	177.9 (2)	C12—C13—C14—O5	−178.3 (2)
C3—C4—C9—C8	−0.3 (3)	C12—C13—C14—C15	0.2 (3)
N1—C5—C9—C4	−177.7 (2)	O5—C14—C15—C16	177.6 (2)
N1—C5—C9—C8	0.6 (3)	C13—C14—C15—C16	−0.7 (3)
N1—C6—C7—C8	1.1 (3)	C14—C15—C16—C6	−178.8 (2)
N1—C6—C7—C10	−179.5 (2)	C14—C15—C16—C17	0.4 (3)
N1—C6—C16—C15	−0.4 (3)	C6—C16—C17—C11	−1.0 (3)
N1—C6—C16—C17	−179.6 (2)	C6—C16—C17—C12	179.6 (2)
C7—C6—C16—C15	179.6 (2)	C15—C16—C17—C11	179.8 (2)
C7—C6—C16—C17	0.3 (3)	C15—C16—C17—C12	0.4 (3)

Symmetry codes: (i) −*x*+1, −*y*+1, −*z*; (ii) −*x*+2, −*y*+1, −*z*; (iii) *x*−1, *y*, *z*; (iv) *x*−1, *y*, *z*−1; (v) *x*, *y*, *z*−1; (vi) *x*, *y*, *z*+1; (vii) *x*+1, *y*, *z*+1; (viii) *x*+1, *y*, *z*; (ix) −*x*+1, −*y*, −*z*+1; (x) −*x*+2, −*y*+1, −*z*+1; (xi) −*x*+1, −*y*, −*z*; (xii) −*x*+2, −*y*, −*z*+1; (xiii) −*x*, −*y*, −*z*; (xiv) −*x*+3, −*y*+1, −*z*+1.
